# Untangling the threads of motivated memory: Independent influences of reward and emotion

**DOI:** 10.3758/s13423-024-02546-9

**Published:** 2024-07-31

**Authors:** Holly J. Bowen, Christopher R. Madan

**Affiliations:** 1 Department of Psychology, Southern Methodist University, PO Box 750442, Dallas, TX 75275-0442 USA; 2 School of Psychology, University of Nottingham, Nottingham, UK

**Keywords:** Emotion, Motivation, Episodic memory, Recognition, Recall

## Abstract

Motivational and emotional influences on memory have been studied extensively; however, despite the link between these constructs, they have been studied in separate lines of research, with very little work examining their effects concurrently. The current study takes a novel approach to manipulate motivational and emotional influences orthogonally, and within the same task, to test their interplay on intentional memory formation. If emotion and reward motivation are tightly linked, they may rely on overlapping cognitive mechanisms, thus we would not expect emotion and reward to interact in memory. Alternatively, they could be distinct constructs and therefore would boost memory when both are included in the same experimental trial, above and beyond additive effects. To test these competing predictions, in Experiment 1, participants (*n* = 180) completed an old/new recognition memory task with emotional (negative, positive) and neutral words intentionally encoded with high or low reward anticipation cues. In Experiment 2, participants (*n* = 159) encoded emotional and neutral words with a high or low reward cue, but memory was tested with free recall using study–test blocks. The findings from both experiments converged. There were main effects of emotion and reward in generally hypothesized directions, but no evidence of an interaction between these factors. This is in line with the prediction that emotion and reward motivation are similar constructs. Their combination within a trial does not boost memory above and beyond either of these factors alone perhaps indicating these constructs have similar cognitive mechanisms.

## Introduction

Our most impactful memories are from events that carry affective significance. These memories, which include both motivational and emotional components, have a pervasive influence on personality, decision-making, and mental health (Madan, 2024; Williams et al., 2022); thus, it is critical to understand how emotion and motivation interactively modulate memory. Motivational and emotional effects on memory have been studied extensively; however, despite the link between these constructs, they have been studied in separate lines of research. Here, we aim to characterize the relationship between emotion, motivation, and memory by studying them together.

Many findings lead one to consider whether emotion and motivation have overlapping mechanisms as their influences on memory performance often mimic each other. Emotional valence impacts objective and subjective aspects of memory formation (for reviews, see Bowen et al., 2018; Clewett & Murty, 2019; Mather & Sutherland, 2009, 2011; Williams et al., 2022). Negative and positive valence can enhance memory accuracy, but, typically, negative information is remembered with greater detail, vividness, and consistency, whereas positive information is remembered in a more gist-like manner (Kensinger, 2009). Like emotion, reward motivation—an impetus for goal-directed behavior—has been shown to both enhance memory (Miendlarzewska et al., 2016; Simonsen & Madan, 2021) and be detrimental to hippocampal-dependent memory (Madan et al., 2017; Murty et al., 2011; Ritchey et al., 2019). An exception to these parallels is for negative affect. Unlike negative emotion—which enhances memory—loss motivation (the opportunity to avoid a loss with task performance) can induce task frustration, greater perceived task demand, and ultimately worse memory performance (Jang et al., 2020). Emotion can also affect response bias at the time of retrieval. Compared with neutral, positive and negative valence may lead to higher false-alarm rates in recognition (Bowen et al., 2016; Kapucu et al., 2008). Similarly, high-reward motivation can also lead to liberal responding at recognition (Bowen et al., 2016).

Pitting emotional and motivational influences against each other at encoding, Eich and Castel (2016) found that high-value items were recalled better than emotionally arousing low-value stimuli. Top-down motivational influences may induce cognitive control during encoding, reducing bottom-up influences of emotional salience on memory, in the service of task goals to maximize points. Gable and Harmon-Jones (2010) compared emotion and motivation and found high-value reward cues narrowed attentional scope and enhanced memory for centrally versus peripherally presented neutral items. A similar pattern was observed when positive images were presented before neutral target items. Building on this, we found that positive affect, whether induced as emotion or reward motivation, had similar effects on recognition, but effects diverged for negative affect (Bowen & Spaniol, 2017).

Few studies have considered the interaction between emotion and motivation within the same trial. In those that have, gain and loss cues are presented before or during the presentation of emotional stimuli, and memory for the emotional stimuli is tested using recognition or recall (for reviews, see Bowen, 2020; Madan, 2017, 2024). Some studies find that congruent valence, the combination of reward cues and emotionally positive, but not negative, stimuli together enhance memory (Mather & Schoeke, 2011; Wittmann et al., 2008; Yan et al., 2023) above and beyond main effects. In these three studies, encoding was incidental to the reward. Participants engaged in a rewarded task within each trial, but successful memory encoding was not tied to the reward (see also Simonsen & Madan, 2021). Reward motivation may impair recognition for emotionally negative stimuli, at least when incidentally encoded (Yan et al., 2023). Two studies (Shigemune et al., 2010; Yan et al., 2017) have tested memory for emotional and neutral stimuli *intentionally* encoded with reward and loss cues that served as incentives for successful memory. Both found main effects of reward and emotion in hypothesized directions, but no interaction. No studies have examined whether these factors interact to influence recognition response bias or free recall rates.

## The current study

If emotion and motivation are tightly linked, they may rely on overlapping mechanisms; thus, we would not expect them to interact in memory. Alternatively, they could be distinct and boost memory above and beyond additive effects. We test these competing predictions in two memory experiments where motivational and emotional influences are manipulated orthogonally, within the same trial.

## Experiment 1

Emotion and reward motivation separately influence memory, but here, we test whether these factors are common or dissociable, additive or interactive, on two signal-detection metrics of recognition. Memory sensitivity is the degree to which one can discriminate target from distractor items, whereas response bias is the tendency to select one response over another (Macmillan & Creelman, 2005). We expected to replicate prior work that, compared with neutral valence, positive and negative valence (Hyp. 1) and high compared with low rewards (Hyp. 2) lead to greater memory sensitivity. Further, we expect similarly valenced *high-reward positive* words will interact and lead to greater memory sensitivity (Hyp. 3), but also to a more liberal “old” response bias compared with low-reward negative words (Hyp. 4).

### Methods

#### Participants

All 202 participants recruited via CloudResearch’s online crowdsourcing platform Connect (Hartman et al., 2023) on February 28, 2023, were located in the United States and restricted to an age range of 18–35 years. Twenty-two participants total were excluded from analyses for being outside the specified age range (*n* = 1), responding to less than 80% of trials during the encoding task (*n* = 2), reporting they wrote down words (*n* = 7), did not pass the attention check (*n* = 4), or reported a neurological disorder or brain injury/insult (e.g., stroke, seizure, electroconvulsive therapy, traumatic brain injury; *n* = 8). An a priori power analysis conducted in G*Power 23 (Faul et al., 2007) indicated a sample of *N* = 78 would provide 95% power to detect a within-subjects effect of f = 0.229, with α = 0.05. This effect size was taken from our study with a similar design (Bowen & Spaniol, 2017).

After exclusions, the final sample included 180 participants (66 female, 113 male, one preferred not to report) ranging from 19 to 35 years old (*M* = 29.5, *SD* = 3.86). Average scores on the Beck Depression Inventory (Beck et al., 1961) were within the minimal range (*M* = 9.86, *SD* = 9.82) but did range from 0 to 47. On average, positive affect (*M* = 28.5, *SD* = 9.38) was higher than negative affect (*M* = 13.33, *SD* = 5.57) as measured with the Positive and Negative Affect Schedule (Watson et al., 1988). Scores on the Behavioral Inhibition Scale (*M* = 20.5, *SD* = 4.76) and Behavioral Activation Scale (Carver & White, 1994) subscales of Drive (*M* = 10.7, *SD* = 2.76), Fun Seeking (*M* = 10.9, *SD* = 2.46), and Reward Responsivity (*M* = 16.6, *SD* = 2.78) were also collected.

Examining other sample characteristics, 86.1% reported they were not and 5% reported they were of Hispanic/Latino/Spanish origin; 6.1% reported Mexican/Mexican American/Chicano origin; 1.7% Puerto Rican origin; < 1% Cuban origin; and < 1% other. Examining race, 67.8% reported White, 12.2% Asian, 12.2% Black/African American, 1.1% American Indian/Alaskan Native, 1.1% Native Hawaiian/Pacific Islander, 1.1% preferred not to disclose, 4.6% reported being of mixed race or other; 58.9% reported a college-level education, 12.2% graduate training, 27.8% high school education, and 0.6% grade school; 79.5% reported being employed full or part time, 6.2% full or part time student, 6.2% homemakers or otherwise not seeking work, and 8.3% were unemployed and seeking work.

Participants were paid $7.50 for participation plus a bonus for performance on the memory task (bonus *M* = $7.54, *SD* = $3.65).

#### Materials

The experiment was programmed and run in Qualtrics (Qualtrics, Provo, UT, USA). A total of 300 words were selected from Warriner et al. (2013) and split into two lists. Each 150-word list contained 50 negative, 50 neutral, and 50 positive words used as target stimuli. Word lists were matched on age of acquisition (overall range = [3.32,16.31]; Kuperman et al., 2012), concreteness ([0, 5]; Brysbaert et al., 2014), (log) frequency ([1.54, 5.53]; van Heuven et al., 2014), and semantic relatedness ([0.80, 0.98]; Mandera et al., 2017). A subset of 75 words (25 negative, 25 neutral, 25 positive) were chosen from each list of 150 to serve as lure stimuli for the other list. This resulted in 225 unique words (150 targets, 75 lures) that were viewed by participants during the experiment. Stimulus assignment to high- or low-reward status was counterbalanced across participants so they were randomly assigned to one of four stimulus lists. The unequal number of target and lure items helped reduce the overall duration of the experiment, an important consideration for online cognitive studies, but participants were not informed about this unequal distribution.

Overall, the average valence ratings for the 100 negative (*M* = 2.54, *SD* = 0.64), 100 neutral (*M* = 5.11, *SD* = 0.42), and 100 positive (*M* = 7.38, *SD* = 0.53) stimuli were all significantly different from each other, *F*(2, 297) = 2010.97, *p* < 0.001. The average arousal ratings for the 100 negative (*M* = 4.88, *SD* = 0.97) and 100 positive (*M* = 4.68, *SD* = 0.94) stimuli were not significantly different from each other (*p* = 0.125), but both were significantly higher than the average arousal ratings for the 100 neutral stimuli (*M* = 4.24, *SD* = 0.72), *F*(2, 297) = 13.07, *p* < 0.001. Comparing the 150 stimuli in the two target list orders, overall they did not differ on ratings of valence, *t*(298) = 0.20, *p* = 0.85, arousal, *t*(298) = 0.73, *p* = 0.47, or on word length, *t*(298) = 0.21, *p* = 0.83. The median word length was seven letters (*M* = 7.24, *SD* = 2.18).

Comparing the 50 negative, 50 positive, and 50 neutral words across list order, ratings did not significantly differ from each other, *t*(98) ≤ 0.72, *p* ≥ 0.47, arousal, *t*(98) ≤ 0.51, *p* ≥ 0.61, or word length, *t*(98) ≤ 0.59, *p* ≥ 0.56 (see Table 1 for means). Negative, positive, and neutral targets did not significantly differ from the negative, positive, and neutral lures within lists on valence, *t*(73) ≤ 1.15, *p* ≥ 0.26, arousal, *t*(98) ≤ 0.76, *p* ≥ 0.45, or word length, *t*(73) ≤ 0.69, *p* ≥ 0.50, (see Table 1 for means) and arousal ratings within list orders.

**Table 1:** Characteristics of the negative, positive, and neutral words for the two stimulus list orders

	Negative List 1	Negative List 2	Positive List 1	Positive List 2	Neutral List 1	Neutral List 2
Target valence	2.51 (0.55)	2.56 (0.72)	7.33 (0.54)	7.41 (0.53)	5.10 (0.44)	5.11 (0.41)
Target arousal	4.93 (0.94)	4.83 (0.99)	4.72 (0.93)	4.65 (0.96)	4.27 (0.72)	4.21 (0.72)
Lure valence	2.61 (0.75)	2.47 (0.60)	7.48 (0.53)	7.26 (0.58)	5.14 (0.48)	5.10 (0.51)
Lure arousal	4.83 (0.99)	4.76 (1.08)	4.64 (0.98)	4.70 (1.02)	4.13 (0.75)	4.28 (0.65)
Target word length (letters)	7.14 (1.93)	7.36 (2.31)	7.32 (2.31)	7.20 (2.12)	7.12 (2.15)	6.86 (2.32)
Lure word length (letters)	6.96 (2.01)	7.04 (1.93)	7.56 (2.22)	6.84 (2.19)	7.04 (2.21)	7.08 (2.48)

Means, standard deviations in parentheses

Three questionnaires were administered during the experiment. The Beck Depression Inventory (BDI; is a 19-item self-report survey of the extent the participant has experienced depressive symptoms over the past 2 weeks. The Positive and Negative Affect Schedule (PANAS) is a mood survey where participants rate 12 positive and 12 negative emotion words on a 5-point scale according to how they feel at that moment. Behavioral Inhibition Scale/Behavioral Activation Scale (BIS/BAS) is a 24-item self-report measure of four facets of the motivational system: Reward Responsivity, Fun Seeking, Drive, and Behavioral Inhibition. Participants also provided demographic information (reported above), and a brief health history (e.g., neurological disorders, psychiatric problems).

#### Procedure

All procedures were approved by the Institutional Review Board at Southern Methodist University. After providing informed consent, participants completed the PANAS, followed by the encoding instructions. Participants were told they were going to study words and their memory for the words would be tested. They were informed that each word they studied would be paired with a reward cue assigned to a high ($0.25) or low ($0.01) value, indicating how much they could earn for successfully recognizing the word on the memory test. Participants were randomly assigned to one of four stimulus lists. Six practice trials were followed by 150 experimental trials presented in random order. During encoding, to keep participants engaged and restrict their ability to write down words, participants made a vowel or consonant judgment of the first letter in each word by pressing the “v” or “c” button on the keyboard. The BDI, BIS/BAS, health, and demographic information were collected after encoding.

Retrieval instructions followed and indicated that studied and nonstudied words would be presented one at a time, and they were to indicate an old/new judgment. They were also informed that in addition to earning rewards for correct recognition, for each “new” item incorrectly judged as “old,” they would lose $0.13. Twelve practice trials (six targets from encoding practice and six lures) were followed by 225 experimental trials (150 targets, 75 lures) presented in random order.

After retrieval, participants were asked whether they wrote words down during encoding to help their memory performance and whether they employed any specific strategies. As an attention check, they were given instructions to select “Other” and type the word “Silver” when asked as a final multiple-choice question “What was the experiment about?” The median time to complete the entire experiment was 35 min and 41 s.

#### Data analysis

Signal detection parameters *d′* and criterion *c* were calculated as measures of memory sensitivity, and response bias, respectively. It is important to note that lures presented at retrieval do not vary by reward level, so the same false-alarm rate was used to calculate these parameters within valence. Each parameter was submitted as a dependent variable in separate 3 (emotional valence: negative, neutral, positive) × 2 (reward: high, low) repeated-measures analysis of variance (ANOVA) using SPSS Version 26. Bonferroni-corrected pairwise comparisons were modeled into the analyses. To determine how null effects should be interpreted, we additionally conducted Bayesian repeated-measures ANOVAs in JASP and report the Bayes factor inclusion (BF_incl_), which quantifies the evidence in the data for including a predictor. BF_incl_ values below 0.33 are suggestive that a model term does not significantly contribute in explaining the data; values below 0.10 are considered moderate evidence against including; values below 0.03 are considered strong evidence against including.

### Results

Examining memory sensitivity* d*’ revealed a main effect of emotion, *F*(2, 358) = 30.75, *p* < 0.001, η_p_^2^ = 0.15. Pairwise comparisons indicated sensitivity was higher for negative (*M* = 0.93, *SE* = 0.05) compared with neutral (*M* = 0.83, *SE* = 0.05), and significantly higher for neutral than positive words (*M* = 0.64, *SE* = 0.05). A main effect of reward *F*(1, 179) = 44.02, *p* < 0.001, η_p_^2^ = 0.20, indicated significantly higher memory sensitivity for high (*M* = 0.91, *SE* = 0.06), compared with low-reward (*M* = 0.69, *SE* = 0.04), words. The Emotion × Reward interaction was not significant, *F*(2, 358) = 2.60, *p* = 0.08, η_p_^2^ = 0.01, BF_incl_ = 0.28.

**Fig. 1:**
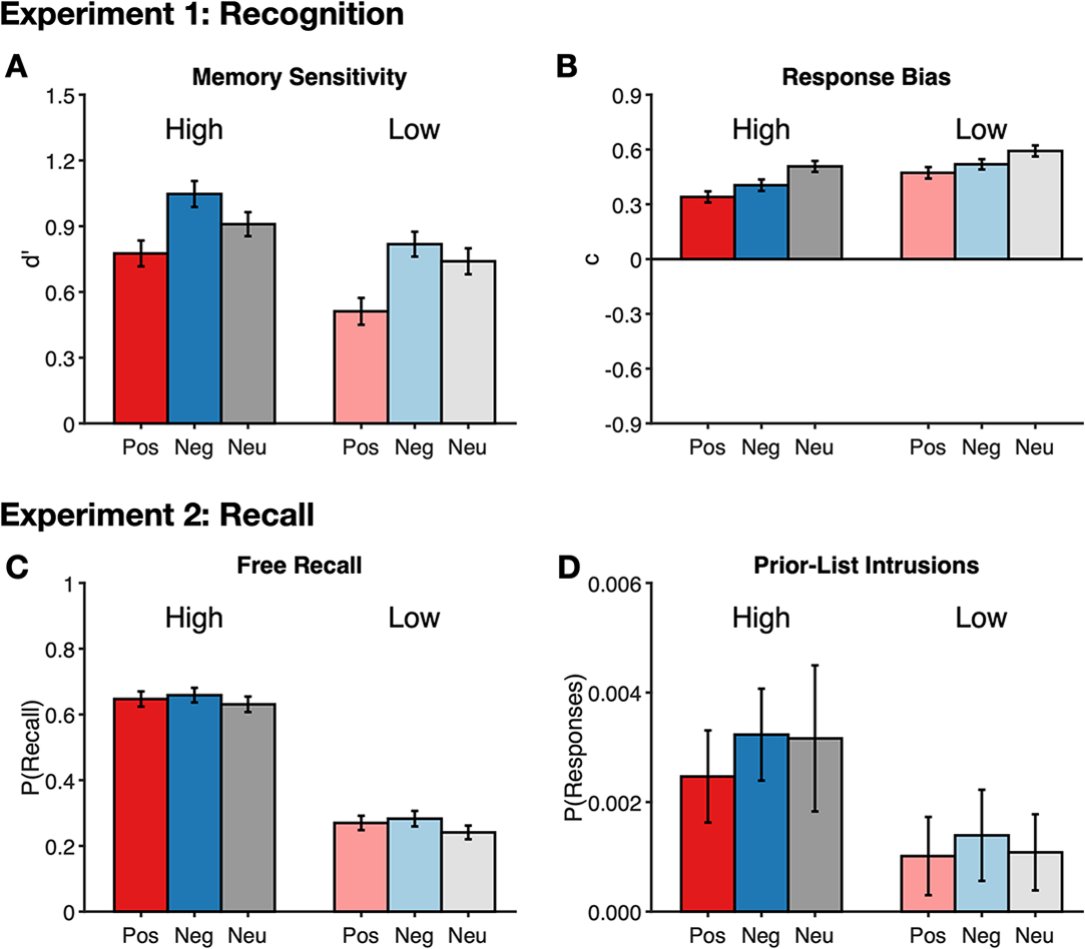
Results for Experiments 1 and 2. Experiment 1 used a recognition procedure, analyzed for (**A**) memory sensitivity, *d′*, and (**B**) response bias, *c*. Experiment 2 used a recall procedure, analyzed for (**C**) free recall and (**D**) prior-list intrusions. For each panel, the left set of bars, shown in darker colors, correspond to the high-reward conditions; the right set of bars, in lighter colors, correspond to the low-reward conditions. Within each set of bars are each emotion valence condition, from left to right, positive (red), negative (blue), and neutral (gray). Error bars are 95% confidence intervals, corrected for interindividual differences (Loftus & Masson, 1994). (Color figure online)

Criterion *c* findings matched *d*′ (see Fig. 1A–B), with a main effect of emotion, *F*(2, 358) = 26.33, *p* < 0.001, η_p_^2^ = 0.13. Pairwise comparisons indicated criterion was less conservative for negative (*M* = 0.46, *SE* = 0.05) and positive words (*M* = 0.41, *SE* = 0.05). compared with neutral words (*M* = 0.55, *SE* = 0.05). All three means were significantly greater than zero, *t*(179) ≥ 8.24, *p* ≤ 0.001, indicating a conservative “new” response bias for negative, neutral and positive words. A main effect of reward *F*(1, 179) = 44.02, *p* < 0.001, η_p_^2^ = 0.20, revealed significantly less conservative responding for high (*M* = 0.42, *SE* = 0.05), compared with low-reward (*M* = 0.53, *SE* = 0.05), words. Again, these means were significantly greater than zero, *t*(179) ≥ 8.98, *p* ≤ 0.001, reflecting a conservative response bias for high and low reward. The Emotion × Reward^1^ interaction was not significant, *F*(2, 358) = 2.60, *p* = 0.08, η_p_^2^ = 0.01, BF_incl_ = 0.26.

## Discussion

We expected an emotional memory enhancement compared with neutral (Hyp. 1), and this was partially supported. Memory sensitivity was higher for negative items, but lower for positive items compared with neutral. Enhanced memory for negative over other valence types aligns with a negativity bias for younger adults (Bowen & Spaniol, 2011; Carstensen & DeLiema, 2018; Dewhurst & Parry, 2000; Kensinger & Schacter, 2006; Norris, 2021; Ochsner, 2000). We also predicted a motivational enhancement (Hyp. 2), and like studies cited in the introduction, memory sensitivity was higher for high- compared with low-reward items.

We additionally predicted an interaction between these factors (Hyp. 3) that similar valence (high-reward positive) would lead to enhanced recognition, but this was not supported. Our final hypothesis (Hyp. 4) that response bias would be more liberal for high-reward positive items was also not supported. Overall, participants were conservative in their responding, meaning more likely to endorse the “new” response across conditions. However, positive and negative items elicited a less conservative criterion than neutral. Findings of a more liberal response bias (more likely to endorse the “old” response) have been previously reported for emotional stimuli (Bowen et al., 2016; Kapucu et al., 2008). High-reward items were found to elicit a less conservative bias than low-reward. The $0.13 false alarm penalty was deducted for each “old” response to lure items to curb liberal responding and prevent “old” responses to all items thus earning all possible rewards. A more conservative response bias for low-reward than high-reward items replicates prior work where inclusion of a false-alarm penalty shifted criterion (Bowen et al., 2020a, 2020b, 2020c).

Criterion may have also shifted because there were unequal numbers of old (150) and new (75) items. This was implemented to decrease study duration. Participants were not informed of these unequal distributions, but metacognitive monitoring may have led participants to infer this information. If this were the case, one might expect a liberal criterion since there are more old than new items, rather than an overall conservative criterion. Further, participants still make “new” judgments even when *only* target items are presented at recognition (Madan et al., 2020).

These findings are in line with prior work discussed in the introduction (Shigemune et al., 2010; Yan et al., 2017). Using an intentional-encoding task, we found main effects of emotion and reward in hypothesized directions, but no interactions, on memory sensitivity or response bias. No interaction may indicate emotion and reward are similar constructs, and their combination within a trial does not boost memory above and beyond either factor alone.

## Experiment 2

While recall and recognition can be affected similarly by item properties and instructions (Lau et al., 2018; Morr, 1981; VanArsdall et al., 2013), this is not always the case (Hall, 1954; Malmberg et al., 2004). Here, we sought to evaluate if the findings we observed with recognition would extend to recall, or if we might yet observe an interaction. We hypothesized the same patterns described for recognition (Hyp. 1–3).

We expected high-reward words to be more retrievable (Hyp. 2), so we examined prior-list intrusions as a second dependent measure. Specifically, does reward-related enhancement of recall result in a greater tendency to falsely recall high- rather than low-reward words in subsequent free-recall blocks (Hyp. 5)? Using a different manipulation, where rewards were learned in an operant-learning task, Madan et al. (2012) found more high-reward intrusions, which were interpreted as an overgeneralization of recall and poor memory control (Miendlarzewska et al., 2016; Talmi et al., 2021).

### Methods

#### Participants

All 198 participants were recruited via CloudResearch’s online crowdsourcing platform Connect (Hartman et al., 2023) on April 4, 2023, were located in the United States and were restricted to an age range of 18–35 years. Participants who completed Experiment 1 were restricted from participating in Experiment 2. Thirty-two participants total were excluded from analyses for being outside the specified age range (*n* = 1), responding to less than 80% of trials on the encoding task (*n* = 6), reporting they wrote down words (*n* = 9), did not pass the attention check (*n* = 5), reported a neurological disorder or major brain injury/insult (e.g., stroke, seizure, electroconvulsive therapy, traumatic brain injury; *n* = 11), or learned English after age 7 (*n* = 1). An additional seven participants were excluded for unreasonably high recall rates (see Data Analysis section for further details).

The final sample after all exclusions was 159 participants (70 female, 87 male, two preferred not to report) ranging in age from 20 to 35 years old (*M* = 29.33, *SD* = 3.94). Average scores on the Beck Depression Inventory (Beck et al., 1961) were within the minimal range (*M* = 9.71, *SD* = 9.86) but did range from 0 to 49. On average, positive affect (*M* = 31.89, *SD* = 9.68) was higher than negative affect (*M* = 13.64, *SD* = 6.35), as measured with the Positive and Negative Affect Schedule (Watson et al., 1988). Scores on the Behavioral Inhibition Scale (*M* = 17.53, *SD* = 3.99) and Behavioral Activation Scale (Carver & White, 1994) subscales of Drive (*M* = 11.18, *SD* = 2.67), Fun Seeking (*M* = 11.01, *SD* = 2.70), and Reward Responsivity (*M* = 16.47, *SD* = 2.73) were also collected.

Examining other sample characteristics, 85.5% reported they were not and 0.6% reported they were of Hispanic/Latino/Spanish origin; 8.2% reported Mexican/Mexican American/Chicano origin; 1.3% Puerto Rican origin; 1.9% some other race, ethnicity, or origin; and 2.6% did not report. Examining race, 65.4% reported White, 10.1% Asian, 16.4% Black/African American, 1.9% American Indian/Alaskan Native, 2.5% preferred not to disclose, 3.7% reported being of mixed race or other; 61.6% reported a college-level education, 11.3% graduate training, 25.2% high school education, and 1.9% grade school; 74.9% reported being employed full or part time, 8.1% full- or part-time student, 6.9% homemakers or otherwise not seeking work, and 10.1% were unemployed and seeking work. Participants were paid $7.50 for participation plus the bonus for performance on the task (Bonus *M* = $12.42, *SD* = $3.83).

#### Materials

The experiment was programmed and run in Qualtrics (Qualtrics, Provo, UT, USA) and included the same stimuli that were used in Experiment 1 list order 1 (Warriner et al., 2013). The list of 150 words was further divided into 10 study–test blocks, each containing five negative, five positive, and five neutral words, and the presentation of the 15 words was randomized across participants. There were two stimulus lists, as assignment to high- or low-reward status was counterbalanced across participants.

#### Procedure

All procedures were approved by the Institutional Review Board at Southern Methodist University. After providing informed consent, participants completed the PANAS, followed by the encoding instructions. Participants were told that on each round they would study 15 words, their memory for the 15 words would be tested after each round, and there would be 10 rounds total. They were informed that each word they studied would be paired with a reward cue of high ($0.25) or low ($0.01) value, indicating how much they could earn for successfully recalling the word on the memory test. After each round of encoding, participants completed the recall task. They were provided with 15 separate text boxes to type answers into and were told they would have up to 1.5 min to recall as many words as possible. Participants could move on to the next round after 6 s by pressing the “next” button if desired. Six study–test practice trials were followed by 10 rounds of experimental trials (150 unique words total). During encoding, to keep participants engaged and restrict the ability to write down words, participants made a vowel or consonant judgment by pressing the “v” or “c” button on the keyboard.

The BDI, BIS/BAS, health, and demographic information were collected after the final recall test. Finally, participants were asked whether they wrote words down during encoding to help their memory performance and whether they employed any specific strategies. As an attention check, they were given instructions to select “Other” and type the word “Silver” when asked as a final multiple-choice question “What was the experiment about?” The median time to complete was 31 min and 6 s.

#### Data analysis

As mentioned in the Participants section, seven participants with more than 130 (out of 150) correct recalls were excluded for unreasonably high recall rates. That is, a recall rate greater than 13 words from a list of 15—this was not affected by the corrections described below, all > 130 responses were typed exactly correctly. Nearly all of these individuals had more than 140 correct recalls (*N* = 6). No participants were excluded for too few recalls (i.e., less than 20 out of 150).

For the retained participants, 10,646 responses were scored as correct (45.2% mean accuracy). An additional 598 responses did not exactly match any words from 150-word study lists. As we are primarily interested in memory—not spelling accuracy—we processed these 598 responses with a spell-checking algorithm (see Madan et al., 2010). Of these, 208 responses were typos (e.g., “acquital”; “dissapointed”; “sophiscation”), 127 were variations stemming from the same root word (e.g., “luxurious” to “luxury”; “fable” to “fabled”), and 201 were valid words corrected to match the study list based on the Levenshtein distance (e.g., “cherry” to “cheery”; “replay” to “repay”). After these corrections, there were 10,715 responses scored as correct (45.5% mean accuracy). The nominal improvement is due to prior-list intrusions, as the process helped match responses to words from the overall study list, not constrained to the associated 15-word list. All subsequent analyses are based on these corrected responses. Prior-list intrusions were any response on the current list that was from a previously presented list (e.g., an intrusion on List 5 could come from any List 1–4). Intrusions were analyzed as proportional to the number of responses provided by the participant (varying by participant; *M* = 71.7, range = [26, 140]). Of these intrusions, 14.7% of intrusions were from the first list presented. We further evaluated the number of intrusions conditional on the current list number, 65.1% were from the immediately prior list and 10.9% were from two lists previous (e.g., for List 5, these would be the percent of intrusions that were from Lists 4 and 3, respectively).

### Results

Mean recall accuracy revealed a main effect of emotion, *F*(2, 312) = 12.64, *p* < 0.001, η_p_^2^ = 0.08. Pairwise comparisons indicated higher recall for negative (*M* = 0.47, *SE* = 0.01) compared with neutral words (*M* = 0.44, *SE* = 0.01), and neutral was significantly lower than positive words (*M* = 0.46, *SE* = 0.01). A main effect of reward, *F*(1, 156) = 358.64, *p* < 0.001, η_p_^2^ = 0.70, indicated significantly better recall for high-reward (*M* = 0.65, *SE* = 0.02), compared with low-reward (*M* = 0.27, *SE* = 0.02) words. The Emotion × Reward interaction was not significant, *F*(2, 312) = 0.69, *p* = 0.50, η_p_^2^ = 0.004, BF_incl_ = 0.17.

Prior-list intrusions revealed a significant main effect of reward, *F*(1, 156) = 14.23, *p* < 0.001, η_p_^2^ = 0.084, with more intrusions of high-reward (*M* = 0.003, *SE* = 0.0004), compared with low-reward (*M* = 0.001, *SE* = 0.0004) words. Neither the main effect of emotion, *F*(2, 312) = 0.69, *p* = 0.50, η_p_^2^ = 0.004, BF_incl_ = 0.02, or the interaction, *F*(2, 312) = 0.22, *p* = 0.80, *η*_p_^2^ = 0.001, BF_10_ = 0.003, were significant. See Fig. 1C–D for a graphical depiction of the means.

### Discussion

We tested whether the findings observed with recognition extend to recall. Hyp. 1 was supported as recall rates were higher for negative and positive words compared with neutral, but there was no difference in recall between negative and positive. This pattern is slightly different than Experiment 1, where recognition was higher for neutral words compared with positive. Like Experiment 1, recall was enhanced for high- compared with low- reward (Hyp. 2) words. We also expected items of similar valence (*high-reward positive)* to interact and lead to greater recall (Hyp. 3), but like Experiment 1, this was not supported. Taken together, findings using a recognition test extend to free recall suggesting these two motivational systems modulate different types of memory retrieval in similar ways.

We expected high-reward words presented in the prior block, to be falsely recalled at higher rates in the subsequent block (Hyp. 5), and this was supported. There were more prior-list intrusions for high- compared with low-reward words aligning with previous work using a different type of reward learning (Madan et al., 2012) and interpreted as reward leading to an overgeneralization of memory, and/or poor cognitive control over memory retrieval at the time of recall. This could also indicate poor source memory, where high-reward items are only weakly associated with temporal context (Miendlarzewska et al., 2016; Talmi et al., 2021). Although there is no penalty for intrusions, high-reward intrusions are competing for cognitive resources to retrieve “valid”, recalls from the current list. Recall of a low-reward word from the current list is beneficial financially, whereas an intrusion of a high-reward word from a prior list provides no additional earnings.

## General discussion

We found clear effects of emotion (Hyp. 1) and reward (Hyp. 2) on memory, but no evidence of an interaction, and this null effect was validated by Bayesian analyses. Memory—whether recognition or recall—is not bolstered by the inclusion of both motivational factors in the same trial, providing robust evidence that emotion and motivation are tightly linked and rely on overlapping mechanisms. Focusing on recognition, both negative emotion and high-reward, enhanced memory sensitivity—the ability to discriminate targets from noise. The false-alarm penalty was successful as responding was conservative overall (favoring the “new” response), but positive and negative emotion and high-reward also led to less conservative responding, a tendency to respond “old” compared with the other conditions. Memory sensitivity and response bias are cognitive mechanisms thought to independently influence performance (Macmillan & Creelman, 2005).

Despite similarities between the patterns in recognition and recall experiments (main effects and no interaction), two interesting differences emerged. First, in recognition, we detected greater memory sensitivity for negative than positive words, but no difference in recall rates or intrusions, highlighting that influences on recall and recognition do not always result in the *exact* same pattern of findings. Second, there were notable differences in the effect size of reward on recognition memory sensitivity (η_p_^2^ = 0.20) versus recall hit rate (η_p_^2^ = 0.70). This could be attributed to several procedural differences across experiments. Recognition involved a single encoding block, and a single recognition block, but recall involved 10 study–test blocks, perhaps leading to greater sensitivity to the reward manipulation. Differences in how accuracy was calculated, and more variability in performance on recall can lead to larger effect sizes if there is a substantial mean difference between experimental tasks. Recognition memory sensitivity takes into account hits and false alarm rates, but recall rate only includes hits, not the prior-list intrusions which were also higher for high- compared with low-reward words (Hyp. 5). This combination of factors might explain the large difference in effect size across experiments.

Higher postlist intrusion for high-reward items could reflect overgeneralization of reward at the time of encoding, and/or reduced cognitive control at retrieval when there is no false-alarm penalty. Our findings from recognition indicate that reward leads to higher memory sensitivity, but an interesting test of overgeneralization would be a rewarded mnemonic similarity test where participants must distinguish between old and new items that are perceptually or conceptually very similar. Finally, high-reward postintrusions could also indicate that reward leads to poor source memory, where high-reward items are only weakly associated with the list/block order or temporal context (Madan et al., 2012; Miendlarzewska et al., 2016; Talmi et al., 2021).

Based on previous studies (Mather & Schoeke, 2011; Wittmann et al., 2008; Yan et al., 2023), we expected positive emotion and reward motivation, conditions of similar valence, to interact (Hyp. 3, 4), but there was no evidence of this. Emotion and reward did not interact in either experiment. An interaction could occur with variations in procedure. First, while emotion is a property of the item, here we used reward as an instruction (similar to Adcock et al., 2006; Bowen, Ford, et al. 2020a, Bowen, Gallant, et al., 2020b, Bowen et al., 2023, Castel et al., 2002; Spaniol et al., 2014). Reward could alternatively be conditioned to be an item property (as in Madan & Spetch, 2012; Madan et al., 2012). By making the motivational factors comparable in this respect, they may be more likely to interact (Bowen, 2020; Madan, 2024). Second, results may differ with more complex tests of memory, such as evaluations of memory specificity (e.g., mnemonic similarity test; Leal & Yassa, 2014; Leal et al., 2014; Swirsky et al., 2020) or composite scenes with central objects and peripheral background elements (Madan et al., 2020). Interactions could occur with an incidental reward-learning task (Madan et al., 2012; Simonsen & Madan, 2021; Wittmann et al., 2008) rather than an intentional encoding task used here. As noted in the introduction, studies where participants earn a reward on each trial, but encoding of the stimuli is incidental to the reward, have shown interactions between positive emotion and high-reward on memory, beyond additive effects.

Interestingly, despite valence differences, negative emotion and reward motivation were similar with respect to the cognitive mechanisms that support memory enhancement, both in recognition and recall. A future experiment to probe these valence effects could benefit from computational models that also incorporate metrics such as reaction time.

## Limitations

Although we replicated expected effects, the studies were conducted online. Attention checks were included to increase and measure task engagement, but there are always concerns about data quality (Gopi et al., 2022). Indeed, some participants were removed from Exp. 2 for very high recall performance. Future studies with in-person samples could confirm these findings. Second, separate false-alarm rates cannot be calculated for each level of reward value because lures are never paired with a reward, but paradigms that permit this calculation would be useful for future research (e.g., Bowen, Marchesi, et al., 2020c), to elucidate the cognitive mechanisms more clearly with computational models. Utilizing a recall task removed response bias but introduced intrusions from previous lists.

## Conclusions

The current studies extend prior work by including both emotion and motivation in the same trial to test for main effects and interactions on recognition and recall memory. Both experiments revealed main effects of emotion and reward in generally hypothesized directions, but no evidence of an interaction. This is in line with the prediction that emotion and reward motivation are similar constructs and rely on overlapping mechanisms such that their combination within a trial does not benefit memory above and beyond either of these factors alone.

## Data Availability

De-identified raw data and study materials are available on our Open Science Framework project page (https://osf.io/hzgte/).

## Footnotes

1 ANOVA statistics for the interactions are the same for sensitivity (*d′*) and response bias (*c*) due to the shared false-alarm rates for high- and low-reward levels.

## Code availability

Analysis code is available on our Open Science Framework project page (https://osf.io/hzgte/).

## Declarations

### Open science practices

De-identified raw data and analysis code are available online (https://osf.io/hzgte/). Neither of the studies was preregistered.
